# The Effect of Eccentric vs. Traditional Resistance Exercise on Muscle Strength, Body Composition, and Functional Performance in Older Adults: A Systematic Review With Meta-Analysis

**DOI:** 10.3389/fspor.2022.873718

**Published:** 2022-04-13

**Authors:** Klemen Čretnik, Jernej Pleša, Žiga Kozinc, Stefan Löfler, Nejc Šarabon

**Affiliations:** ^1^Faculty of Health Sciences, University of Primorska, Izola, Slovenia; ^2^Andrej Marušič Institute, University of Primorska, Koper, Slovenia; ^3^Ludwig Boltzmann Institute for Rehabilitation Research, St. Pölten, Austria; ^4^Human Health Department, InnoRenew CoE, Izola, Slovenia; ^5^Laboratory for Motor Control and Motor Behavior, S2P, Science to Practice, Ltd., Ljubljana, Slovenia

**Keywords:** elderly, sarcopenia, eccentric training, eccentric exercise, older adults

## Abstract

The effects of eccentric exercise (ECC) in older adults have received limited scientific attention, considering the ample evidence for its effectiveness in general and athletic populations. The purpose of this paper is to review the effects of ECC exercise modalities vs. traditional or concentric (CON) exercise on muscle strength, body composition and functional performance in older adults. Inclusion criteria regarding the age was >55 years. Three major scientific literature databases (PubMed, Scopus and Web of Science) were screened for trials comparing the effect of ECC and CON exercise programs, and 19 papers were included in the meta-analysis. ECC and CON training programs were typically matched by the duration of each session. The difference between ECC and CON was expressed as standardized mean difference (SMD). Regarding isometric knee strength, the pooled effect favored ECC (SMD = 0.50), but was not statistically significant (*p* = 0.160). ECC exercise elicited greater improvements in timed up and go test (SMD = −0.68; *p* = 0.004), 2-min sit-stand test (SMD = 0.53; *p* = 0.030) and 30-s sit-stand test (SMD = 0.81; *p* = 0.002), but not in 6-min walking test (SMD = 0.01; *p* = 0.960). The effects on body composition and muscle architecture were unclear (SMD = −1.44 to 1.95; *p* = 0.060–0.689). In conclusion, our literature review indicates that ECC exercise is superior to, or at least as good as CON exercise for preserving health and overall function in older adults.

## Introduction

Aging is associated with numerous physiological and morphological changes, related to declines in functional abilities and susceptibility to age-related diseases, which negatively influences the quality of life and independency (Deschenes, 2004; Capodaglio et al., 2005). Exercise interventions have been shown to improve cardiovascular health, decrease occurrence of sarcopenia and decrease fragility and risk of falling (Macaluso and De Vito, 2004; Benichou and Lord, 2016). Structured exercise is regarded as an effective method to prevent, delay, or attenuate the effect of aging, especially in terms of functional ability and physiological changes (Viña et al., 2016).

A plethora of factors needs to be considered in exercise prescription. One of such important factors is the type of muscle contraction. Muscular actions during resistance exercise may be isometric (i.e., the muscle length is maintained during activation), concentric (i.e., the muscle shortens during activation) and eccentric (i.e., the muscle lengthens during activation), all of which present distinct physiological, neural and mechanical characteristics and responses (Peake et al., 2005; Raman et al., 2012). It has been argued that any training regime that maximizes muscle strength will ultimately improve functional abilities (Malbut-Shennan and Young, 1999). On the other hand, many studies indicate that eccentric-focused training on eccentric ergometer promotes greater gains in muscle strength and hypertrophy when compared to conventional strength training (slow concentric and eccentric actions) performed in “standard” training regime of 10 repetitions per set (LaStayo et al., 2003; Mueller et al., 2009). Furthermore, LaStayo et al. (2014) reported that low-intensity endurance-focused eccentric contractions in eccentric cycling can result in large muscle hypertrophy in older adults. On the other hand, the study by Lewis et al. (2018) showed that the strength gains may not differ between training modalities (ECC and CON cycling) when the intensities are matched in middle-aged sedentary males. Nevertheless, this information should be carefully considered, because the participants in the study are middle age males, which means that the results could be different for older adults. Moreover, Gault and Willems (2013), reported that endurance eccentric exercises (e.g., eccentric cycling, downstairs walking) are adequate for elderly adults to reduce risk of falls and to improve their quality of life. It is also important to note that many tasks that are associated with a high risk of falling, such as descending stairs, relay heavily on eccentric muscle contractions.

In addition to superior effect of eccentric over traditional resistance exercise regarding strength and hypertrophy adaptations, studies reporting lower rate of perceived effort in eccentric compared concentric exercise (Lindstedt et al., 2001; LaStayo et al., 2003). Similar results have been shown for eccentric cycling compared to concentric cycling at the same intensity at given heart rate or oxygen consumption (LaStayo et al., 1999; Peñailillo et al., 2014; Clos et al., 2019). The most important drawback of eccentric exercise is a the possibility of exercise-induced muscle damage (Hody et al., 2013; Jamurtas et al., 2013). Moreover, eccentric exercise is sometimes somewhat more difficult to implement in contrast to conventional exercise methods because a) of safety reasons (eccentric exercises are usually performed at higher intensity, thus it is recommended that exercise is performed with the help of exercise practitioner or “spotter”) and b) lack of appropriate equipment. Given that both advantages and drawbacks of eccentric exercise have been identified, investigating the effect of eccentric-focused exercise in contrast to conventional resistance exercise is important to provide practitioners with optimal and comprehensive guidelines.

In contrast to general and athletic populations, older adults have received less scientific attention in relation to the effect of eccentric exercise. The effect of exercise interventions in older adults has been evaluated through different testing methods, such as functional ability tests (timed-up-and go test (TUGT), 5-repetition sit-to-stand, walking speed, etc.), body composition (muscle mass, fat mass, muscle cross-section area, etc.) and tests of motor abilities (e.g., strength, endurance, balance, etc.). Performance in functional tasks such as TUGT and 5-repetition sit-to-stand can be used to predict the risk for recurrent falls (Buatois et al., 2008). Moreover, performance of everyday functional tasks such as crossing the road or carrying bags from the store are also, to some extent, associated with isokinetic strength (Doherty, 2003). Two very recent systematic reviews focused on the effect of eccentric training in healthy older adults (Molinari et al., 2019; Kulkarni et al., 2021). Molinari et al. (2019) included 5 studies with muscle strength outcomes, and reported similar effects of eccentric exercises and traditional resistance exercises, with the data slightly favoring the former. Kulkarni et al. (2021) examined 10 studies and reported that eccentric exercises can be as effective as conventional exercises in older adults for improving functional performance. In sum, existing reviews are showing the potential of eccentric exercise to elicit similar improvement in comparison to traditional resistance exercise. In light of advantages of eccentric exercise, such as lower energy expenditure, it could be suggested that eccentric exercise should be incorporated in resistance exercise programs for older adults. However, only limited number of variables have been included in existing reviews, thus further analysis is needed to provide broader view about the effects of eccentric exercise and eccentric training modalities compared to traditional training type on basic motor capabilities, body composition and functional ability in older adults. Based on that, the objective of this paper is to review the effects of eccentric exercise modalities vs. traditional resistance exercise on muscle strength, body composition and functional performance in older adults. In accordance with the previous evidence, we hypothesized that eccentric exercise will have similar effect on functional performance, muscle strength and body composition, compared to traditional resistance exercise.

## Methods

### Search Strategy

The search was performed in October 2021. Three major scientific literature databases (PubMed, Scopus and Web of Science) were screened, using the following search term: (eccentric exercise OR flywheel OR isoinertial exercise OR eccentric training) AND (older adults OR elderly OR elders OR old age OR aging). The records were imported into Mendeley (version 1.19.8) to remove the duplicates, and then exported into Microsoft Excel software. The search strategy was carried out in three stages: (1) assessing the eligibility of the papers based on the title, (2) assessing the eligibility of the papers based on the abstract and (3) assessing the eligibility of the papers based on the full text. Both reviewers assess all papers. At all three stages, two reviewers carried out the procedures independently. In case of non-agreement in stages 1.-2., any papers that were identified by only one reviewer were carried over to the next phase. In stage 3., potential disagreements were resolved by additional discussion and consultation of the third reviewer.

### Inclusion Criteria

The inclusion criteria are structured according to the PICOS tool (Methley et al., 2014), as follows:

- **P (population):** older adults, aged > 55 years. We excluded patients with neurological diseases (e.g., Parkinson's disease), but we also considered patients with metabolic and cardio-vascular diseases.
- **I (Intervention):** Resistance exercise interventions focusing on (or emphasizing) eccentric contraction. This includes flywheel training and functional tasks such as loaded stair descent. Duration of the intervention > 4 weeks.
- **C (Comparison):** Resistance exercise interventions, performed in a traditional manner or emphasizing concentric contraction.
- **(Outcome):** Outcomes describing muscle performance (muscle strength or power), body composition outcomes (e.g., lean mass, muscle thickness, body fat mass) and functional performance tests (e.g., 6-min walking test, sit-stand tests, stair walking, etc.).
- **S (Study design):** Interventional clinical trials, with at least two groups (eccentric and traditional/concentric exercise groups).

### Data Extraction

The data extraction was carried out independently by two reviewers and disagreements were resolved through consultation with other reviewers. The extracted data included: (a) baseline and post-intervention means and standard deviations for all eligible outcome measures for eccentric and concentric groups; percent changes were considered instead of pre-post data when available (b) baseline demographics of participants (gender, age, body height, body mass, body mass index); (c) intervention characteristics (target body area (upper, lower or whole-body), duration of the intervention, number of sessions per week, volume (number of exercises, sets, and repetitions), breaks between exercises and sets, supervision, and progression of exercise difficulty). Data were carefully entered into Microsoft Excel 2016 (Microsoft, Redmond, WA, USA). If the data were presented in a graphical rather than tabular form, we used Adobe Illustrator Software (version CS5, Adobe Inc., San Jose, CA, USA) to accurately determine the means and standard deviations. In case of missing data, the corresponding author of the respective article was contacted by e-mail. If no response was received after 7 days, the author was contacted again. If the author did not reply to the second inquiry, the data was considered irretrievable.

### Assessment of the Quality of the Included Studies

Two reviewers evaluated the quality of the included studies using the PEDro scale (Maher et al., 2003), which assesses study quality based on a 0–10 scale. Potential disagreements between the reviewers were resolved by consulting the other authors. Studies scoring from 9 to 10 were considered as “excellent,” 6 to 8 as “good,” 4 to 5 as “fair,” and <4 as “poor” quality. The PEDro scale was selected because it was developed to assess the quality of clinical trial studies evaluating physical therapy interventions.

### Data Analysis

The main data analyses were carried out in Review Manager (Version 5.3, Copenhagen: The Nordic Cochrane Center, The Cochrane Collaboration, London, UK). Before the results were entered into the meta-analytical model, the pre-post differences and pooled standard deviations were calculated according to the following formula SD = √[(${\text{SD}}_{\text{pre}}^{2}$ + ${\text{SD}}_{\text{post}}^{2}$) – (2 × r × SD_pre_ × SD_post_). The correction value (r), which represents the pre-test–post-test correlation of outcome measures, was conservatively set at 0.75. It should be noted that a change in the correction value in the range between 0.5 and 0.9 had little effect on the pooled SD and would not change the outcomes of the meta-analyses. For the meta-analysis, the inverse variance method for continuous outcomes with a random-effects model was used. The effect sizes were expressed as standardized mean difference (SMD). For SMD, the respective 95% confidence intervals were also calculated and reported.

The analysis compared the effects of the eccentric exercise and traditional resistance exercise or concentric exercise interventions. Statistical heterogeneity among studies was determined by calculating the *I*^2^ statistics. According to Cochrane guidelines, the *I*^2^ statistics of 0% to 40% might not be important, 30 to 60% may represent moderate heterogeneity, 50–90% may represent substantial heterogeneity, and 75–100% indicates considerable heterogeneity. The threshold for statistical significance was set at *p* ≤ 0.05 for the pooled effect size. Sensitivity analysis was performed by examining the effect of exclusion of studies one-by-one from the analyses.

## Results

### Search Summary

**Figure 1:**
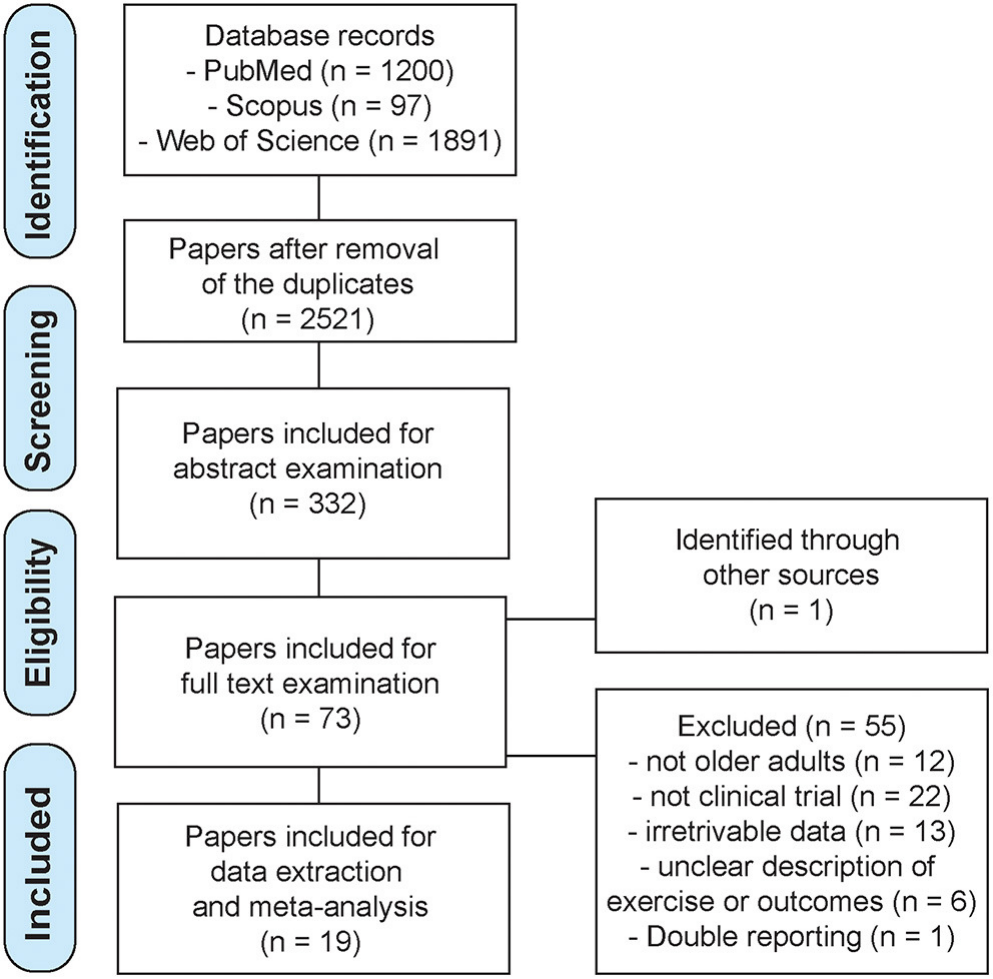
Flowchart with search protocol summary.

The initial search yielded 3,188 records (PubMed = 1,200; Scopus = 97; Web of science = 1,891). After the duplicates were removed, 2,521 records were left for examination. Based on the title, Reviewer 1 identified 264 potentially relevant papers, and Reviewer 2 identifies 242. Most of the papers were overlapping between the reviewers, thus, the sum of the identified papers was 332. In the next step, based on the abstract reading, Reviewer 1 identified 61 potentially relevant papers, and Reviewer 2 identified 57. At this stage, the reference lists of relevant reviews were also scrutinized, and 1 additional paper was included. In total, 73 papers were included for a full-text examination. Both reviewers identified 18 eligible papers with a complete agreement, thus, 19 papers were included into the meta-analysis. The search is summarized on the Flowchart in Figure 1. Supplementary Table 1 also includes the list of papers included at each stage, as well as the extracted data. Table 1 includes basic information regarding the included studies.

**Table 1:** Overview of the studies included into meta-analyses.

**Study**	**Intervention**	**Basic participant data**	**Exercise type**	**Outcome**	**Main conclusion**
Casillas et al., 2016	7 weeks, 3 times/week, 32 min/session	*n* = 42 (21 ECC; 21 CON) Age = 63.7 ± 10.1 ECC; 61.83 ± 8.5 CON BMI = 25.4 ± 3.8 ECC; 25.8 ± 3.7 Height = 171.1 ± 6.7 ECC; 170.4 ± 8.2 CON Weight = 74.5 ± 13.1 ECC; 75.2 ± 13.2 CON	ECC cycling vs. CON cycling	Isometric quadriceps torque, isometric triceps surae torque, peak work rate, 6MWT	Improvement in peak work and 6MWT was the same for the both groups. Maximal strength in triceps surae was increased only in ECC group.
Katsura et al., 2019	8 weeks, 3 times/week, 90 min/session	*n* = 17 (9 ECC; 8 CON) Age = 72 ± 6.6 ECC; 71.1 ± 4.5 CON BMI = 23.5 ± 4.5 ECC, 22.9 ± 1.5 CON Height = 160.8 ± 10.9 ECC; 155.5 ± 5.5 CON Weight = 60.3 ± 9.8 ECC; 55.4 ± 5.4 CON	ECC whole body exercises vs. CON whole body exercises	Isometric quadriceps torque, 30 s chair stand test, TUG, muscle volume of quadriceps femoris, 2 min step test, sit and reach test	ECC training was more effective for improvement of lower limb strength, mobility, muscle volume and postural stability in older adults when compared with CON training.
LaStayo et al., 2009	12 weeks, 3 times/week, 30 min/session	*n* = 17 (9 ECC; 8 CON) Age = 67 ± 8.8 ECC; 68 ± 8.7 CON BMI = 32.4 ± 4.4 ECC; 33.5 ± 5.4 CON	ECC lower body exercises vs. CON lower body exercises	Isometric quadriceps torque, 6MWT, TUG, muscle volume of quadriceps femoris, stair descent, stair ascent	Increase in muscle size and strength and improvement in functional ability tasks were greater in ECC group.
Mueller et al., 2009	12 weeks, 2 times/week, 35–45 min/session	*n* = 46 (23 ECC; 23 CON) Age = 80.3 ± 0.7 ECC; 80.1 ± 0,8 CON BMI = 23.5 ± 0.6 ECC; 24.3 ± 1 CON Height = 168 ± 2 ECC; 167 ± 2 CON Weight = 66.1 ± 1.8 ECC; 67.7 ± 2.6 CON	ECC ergometer training vs. conventional resistance training	Isometric quadriceps torque, TUG, Berg balance scale, Total fat mass	Maximal isometric leg extension strength, loss of body fat and thigh fat were significantly improved only in ECC group. Authors concluded that both ECC and CON training are beneficial for the elderly with regard to muscle function and structural improvements.
Mueller et al., 2011	12 weeks, 2 times/week, 40 min/session	*n* = 27 (13 ECC; 14 CON) Age = 80.3 ±1 ECC; 79.9 ± 1 Height = 168 ± 2.4 ECC; 169.8 ± 2.4 CON Weight = 68.5 ± 2.7 ECC; 74.9 ± 3.4 CON	ECC ergometer training vs. conventional resistance training	Isometric quadriceps torque, Thigh mass, Total fat mass	Both ECC and CON training regimes showed similar functional improvements, with ECC group being at least as effective or more effective as CON group.
Quinlan et al., 2021	8 weeks, 3 times/week, 60 min/session	*n* = 17 (9 ECC; 8 CON) Age = 67.5 ± 1.5 ECC; 69.1 ± 3.0 CON BMI = 24.7 ± 2.9 ECC; 25.7 ± 2.2 CON Height = 176 ± 8.8 ECC; 175 ± 3.3 CON Weight = 76.8 ± 10.4 ECC; 79.2 ± 8.5 CON	ECC leg press vs. CON leg press	Isometric quadriceps torque	Strength increase was similar between both groups.
Reeves et al., 2009	14 weeks, 3 times/week, 10 min/session	*n* = 19 (10 ECC; 9 CON) Age = 67 ± 2 ECC; 74 ± 3 CON Height = 168 ± 12 ECC; 163 ± 10 CON Weight = 74 ± 15 ECC; 70 ± 16 CON	ECC knee extension vs. CON knee extension	Volume of quadriceps femoris, Knee extensor pennation angle, knee extensor fascial length	Increase of fascial length was greater in ECC group. conversely, pennation angle significantly increased only in CON group. In ECC group, eccentric muscle strength was increased, with no change in concentric torque, and vice versa for CON group (increased concentric torque with no change in eccentric torque). Isometric torque increased to a similar extent in both groups.
Regnersgaard et al., 2021	6 weeks, 3 times/week, 45–60 min/session	*n* = 21 (7 ECC; 7 ECC*, 7 CON) Age = 71 ± 1 ECC; 70 ± 1 ECC*; 70 ± 1 CON BMI = 27 ± 0.7 ECC; 25.4 ± 1.7 ECC*; 24.8 ± 2 CON Weight = 73.8 ± 3.1 ECC; 73.4 ± 7 ECC*; 65.1 ± 6.6 CON	Stair ascent vs. Stair descent vs. Stair descent with 15% of persons additional weight (ECC*)	6MWT, 30 s chair stand test, Thigh mass	Leg muscle mass increased more in ECC*and ECC compared to CON. 6MWT and 30 s chair stand test increased more in ECC*compared to other groups. Carrying extra weight while descending star walking do not increase RPE, but still resulted in greater responses compared with CON.
Steiner et al., 2004	8 weeks, 3 times/week, 30 min/session	*n* = 12 (6 ECC; 6 CON) Age = 55 ± 2.6 ECC; 56 ± 3.5 CON BMI = 27.6 ± 1.3 ECC; 27.1 ± 1.9 CON Height = 173 ± 3.9 ECC; 169 ± 0.8 CON Weight = 83 ± 8.1 ECC; 77 ± 1.9 CON	ECC ergometer training vs. standard cycle ergometer	Isometric quadriceps torque, Quadriceps lean tissue cross section area	Muscle mass increased significantly in both groups. Strength parameters improved only in ECC group, while fiber size increased only in CON group.
Symons et al., 2005	12 weeks, 3 times/week, 30 min/session	*n* = 19 (9 ECC; 10 CON) Age = 70.5 ± 5.2 ECC; 71,8 ± 3.1 CON Height = 166.1 ± 9.1 ECC; 168.9 ± 10.3 CON Weight = 80.7 ± 11.6 ECC; 78.5 ± 11.2 CON	ECC isokinetic training vs. CON isokinetic training	Isometric quadriceps torque	Both groups were effective in increasing strength and improving stair-climbing performance. ECC training was not superior to CON training.
Theodorou et al., 2013	6 weeks, 3 times/week, 12 min/session	*n* = 12 (6 ECC; 6 CON) Age = 66.8 ± 1.7 ECC; 64.8 ± 2.3 CON Weight = 85.8 ± 3.1 ECC; 82.1 ± 2.3 CON	Stair ascent vs. Stair descent	Isometric quadriceps torque	Both groups increased muscle strength. Stair descending appears to be less demanding than stair ascending, while changes in muscle strength are similar or even greater.
Besson et al., 2013	7 weeks, 3 times/week, 32 min/session	*n* = 30 (15 ECC; 15 CON) Age = 63.3 ± 10.1 ECC; 60.7 ± 11.8 CON Height = 170.9 ± 7.8 ECC; 170 ± 7 CON Weight = 70.3 ± 15.6 ECC; 74.7 ± 14.5 CON	ECC cycling vs. CON cycling	6MWT	Distance in 6MWT improved in both groups with ECC group being slightly better. ECC training induces functional improvement similar to conventional training, with lower demand on the cardiovascular system during exercise.
Bourbeau et al., 2020	10 weeks, 3 times/week, 30 min/session	*n* = 20 (10 ECC; 10 CON) Age = 68.5 ± 5.6 ECC; 65.2 ± 5 CON BMI = 24.9 ± 5.4 ECC; 25.8 ± 5.8 CON Height = 170.9 ± 10 ECC; 170 ± 8 CON Weight = 73.2 ± 20.4 ECC; 74.6 ± 18.3 CON	ECC cycling vs. CON cycling	Isometric quadriceps torque, 6MWT, stair ascent, steps per day	Muscle strength improved only in ECC group. Training-induced improvement for the 6MWD was observed only in the ECC group. Improvement in the total number of daily step counts from baseline were observed only after ECC training.
Chen et al., 2017a,b	12 weeks, 2 times/week, 5–60 min/session	*n* = 30 (15 ECC; 15 CON) Age = 66.4 ± 6.8 ECC; 66.5 ± 6.8 CON BMI = 26.2 ± 1.1 ECC; 26.1 ± 0.8 CON Height = 155.2 ± 5.8 ECC; 155.2 ± 5.8 CON Weight = 62.7 ± 6.4 ECC; 62.8 ± 5.1 CON	Stair ascent vs. Stair descent	Isometric quadriceps torque, 6MWT, 30 s chair stand test, TUG, 2-min step test, Thigh mass, Body mass	Muscle strength increased more in ECC group. Moreover, results of many functional ability tests show significantly greater improvement for ECC group compared to CON group.
Chen et al., 2017a	12 weeks, once per week, 10–100 min/session	*n* = 26 (13 ECC; 13 CON) Age = 65.9 ± 4.7 ECC & CON BMI = 25.5 ± 2.9 ECC & CON Height = 164.7 ± 5 ECC & CON Weight = 70.5 ± 8 ECC & CON	ECC knee extension vs. CON knee extension	Isometric quadriceps torque, 6MWT, 30 s chair stand test, TUG, 2-min step test, Thigh mass, Postural balance	Muscle strength and functional ability performance (e.g., 30 s chair stand, TUGT, balance) improved greater in ECC group compared to CON group. Eccentric exercise training was more effective than concentric exercise training to improve health and functional fitness in older adults.
Gault et al., 2012	12 weeks, 3 times/week, 30 min/session	*n* = 26 (13 ECC; 11 CON) Age = 67 ± 4 ECC & CON BMI = 26.6 ± 3.9 ECC & CON Height = 170 ± 9 ECC & CON Weight = 77.2 ± 13.9 ECC & CON	Downhill treadmill walking vs. level treadmill walking	TUG	Improvements in functional ability tests were substantial and similar in both groups.
Gluchowski et al., 2017	8 weeks, 2 times/week	*n =* 22 (11 ECC; 11 CON) Age = 67 ± 4.5 ECC & CON Height = 169 ± 8.6 ECC & CON Weight = 75 ± 15.3 ECC & CON	ECC leg press vs. CON leg press	Stair descent, Leg soft tissue lean mass, Total fat mass	Muscle strength and functional ability tasks equally improved in all groups. Body composition significantly improved only in ECC group, with no statistical differences between groups (ECC, CON, eccentric based group).
Onambélé et al., 2008	12 weeks, 3 times/week	*n =* 24 (12 ECC; 12 CON) Age = 69.6 ± 1.1 ECC; 70.2 ± 1.5 CON BMI = 24.3 ± 2 ECC, 24.1 ± 2.5 CON	Flywheel VS inertial load machine	Isometric quadriceps torque, triceps surae maximal isometric torque, eyes open ML sway, eyes open AP sway	ECC group significantly increased quadriceps and triceps surae strength, while CON group increased only triceps surae strength. ECC training results in greater improvements in muscular strength and balance compared to CON group.
Jacobs et al., 2014	12 weeks, 3 times/week, 60 min/session	*n =* 77 (39 ECC; 38 CON) Age = 76.2 ± 7.4 ECC; 74.6 ± 6.2 CON BMI = 27.1 ± 4.8 ECC; 29.1 ± 6.2 CON	ECC ergometer stepper vs. Leg press and straight leg exercise	Leg soft tissue lean mass	No differences in intermuscular adipose tissue (IMAT) were observed over time, and there were no differences in IMAT response between intervention groups. Moreover, participants in CON group lost a significant amount of lean tissue in the 9 months after intervention, while participants in the ECC group did not.

*ECC, eccentric exercise; CON, concentric exercise; BMI, body mass index; 6MWT, six-min walking test; TUGT, timed up and go test*.

### Assessment of Study Quality

All of the studies received the PEDro score in the 3–6 range. Two studies received a score of 3 points, indicating “poor” quality. One study received the score of 6, indicating “good” quality. The remaining studies were of “fair” quality (9 studies with a score of 4; 7 studies with a score of 5). The mean PEDro score was 4.4 ± 0.8. Overall, the evidence presented in this review may be considered to be of “fair” quality. The most common items that almost all studies failed to satisfy were blinding of the subjects, therapists and assessors. Exercise intensity and type of the exercise (whole body only or single-joint task) were not part of the sorting criteria.

### Muscle Strength

**Figure 2:**
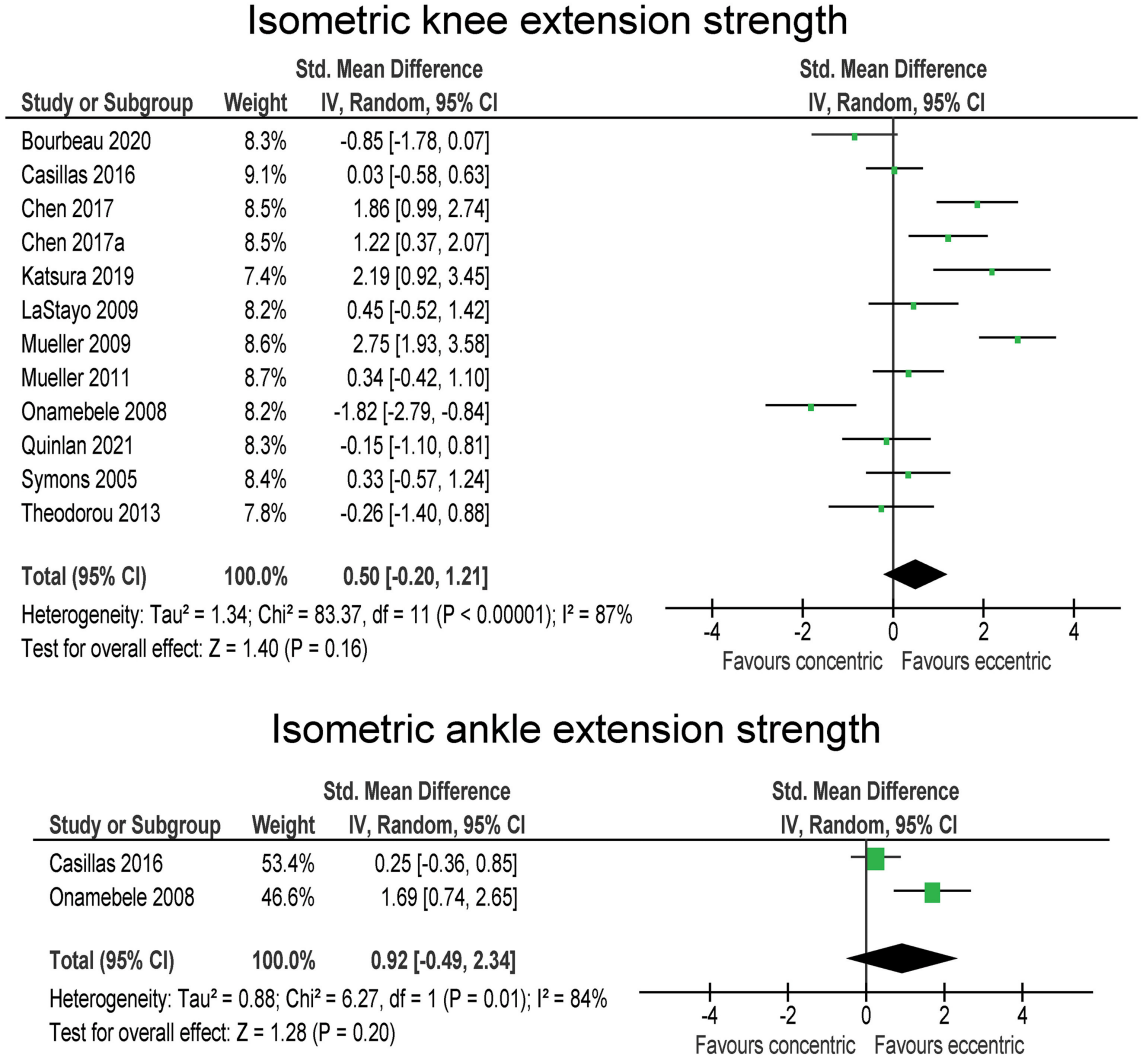
The effect of eccentric vs. concentric exercise on muscle strength outcomes.

Isometric muscle strength assessments were included in 12 studies, involving 149 participants in eccentric exercise groups and 148 participants in traditional resistance or concentric exercise groups (Figure 2). The overall effect, although favoring the eccentric exercise group (SMD = 0.50), was not statistically significant (*p* = 0.160). Moreover, the heterogeneity across the studies was high (*I*^2^ = 87 %). Sensitivity analysis showed that the exclusion of one study that favored concentric exercise (Onambélé et al., 2008) swayed the pooled effect enough to be statistically significant (*p* = 0.030) in favor of eccentric exercise (SMD = 0.71). Isometric ankle extension strength was assessed in two studies (with 33 participants in total for each group). The results are in favor of eccentric exercise with moderate effect (SMD = 0.92), but the difference between the exercise types was not statistically significant (*p* = 0,200), and the two studies were very heterogeneous (*I*^2^ = 84 %) (Figure 2).

### Body Composition and Muscle Architecture

**Figure 3:**
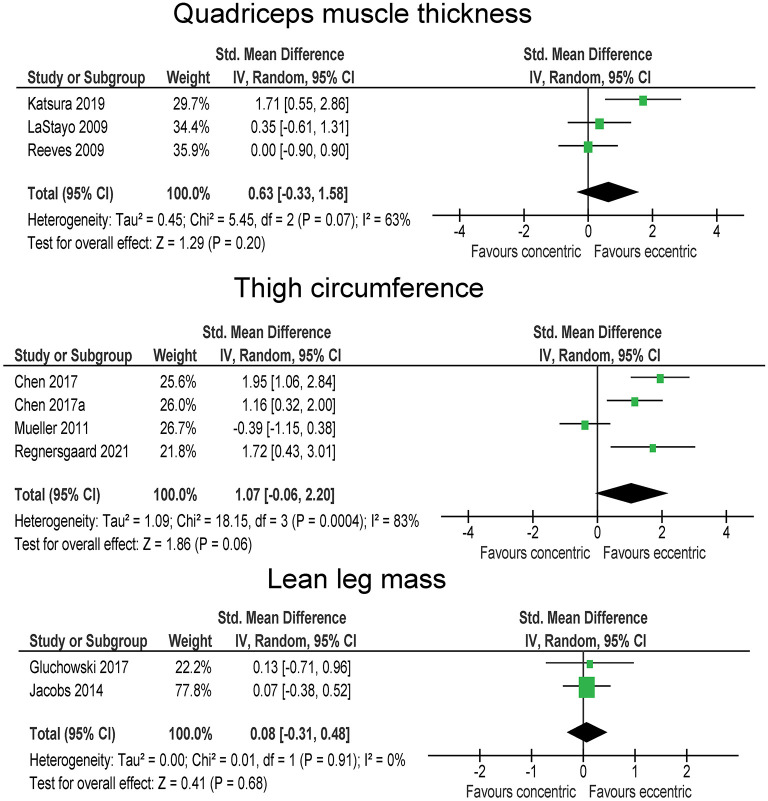
The effect of eccentric vs. concentric exercise on outcomes related to body composition.

Body fat percentage and body fat mass were considered in three studies, with a total number of 47 participants in eccentric exercise groups and 48 participants in in traditional resistance or concentric exercise groups Although the overall effect was large (SMD = −1.44) and showing decreases in body fat with eccentric exercise, it was statistically not significant (*p* = 0.220). A closer inspection of the data revealed that two studies showed almost no difference between the exercise types, while one study pointed heavily toward fat-lowering effect of eccentric exercise in comparison to traditional resistance or concentric exercise. Accordingly, the heterogeneity between the studies was very high (*I*^2^ = 95 %). Lean leg mass was reported in two studies (with 50 and 49 participants in total for eccentric and concentric/traditional exercise groups, respectively) (Figure 3). The difference between the exercise modes was negligible (SMD = 0.08; *p* = 0.680).

Four studies compared the effect of eccentric and traditional resistance or concentric exercise on quadriceps muscle thickness (28 and 25 participants in total within eccentric and concentric/traditional groups, respectively) (Figure 3). The overall effect tended to support the superiority of eccentric exercise (SMD = 0.63), but was not statistically significant (*p* = 0.200) and the heterogeneity among the studies was high (*I*^2^ = 63 %). The results for thigh circumference and thigh mass were pooled together from four studies, involving a total of 48 and 49 participants in eccentric and traditional resistance or concentric exercise groups, respectively. Three studies indicated larger thigh circumference/mass increases in eccentric group with large effect sizes (SMD = 1.16–1.95), however, one study pointed in the opposite direction (SMD = −0.39), yielding a large pooled effect that tended toward better effects in eccentric groups (SMD = 1.07), but slightly above the threshold for statistical significance (*p* = 0.060). The heterogeneity among the studies was high (*I*^2^ = 63%) (Figure 3), with three studies favoring eccentric and one study favoring traditional resistance. In a sensitivity analysis, the exclusion of the latter study (Mueller et al., 2011) resulted in statistically significant effect (*p* < 0.001) favoring eccentric exercise (SMD = 1.57). One study also involved the measurement of pennation angle and fascicle length of the vastus lateralis muscle. There was a large effect in favor of traditional resistance or concentric exercise for pennation angle (SMD = 2.65), while the opposite was true for the fascicle length (SMD = 1.25) (both *p* < 0.01).

### Mobility and Function

**Figure 4:**
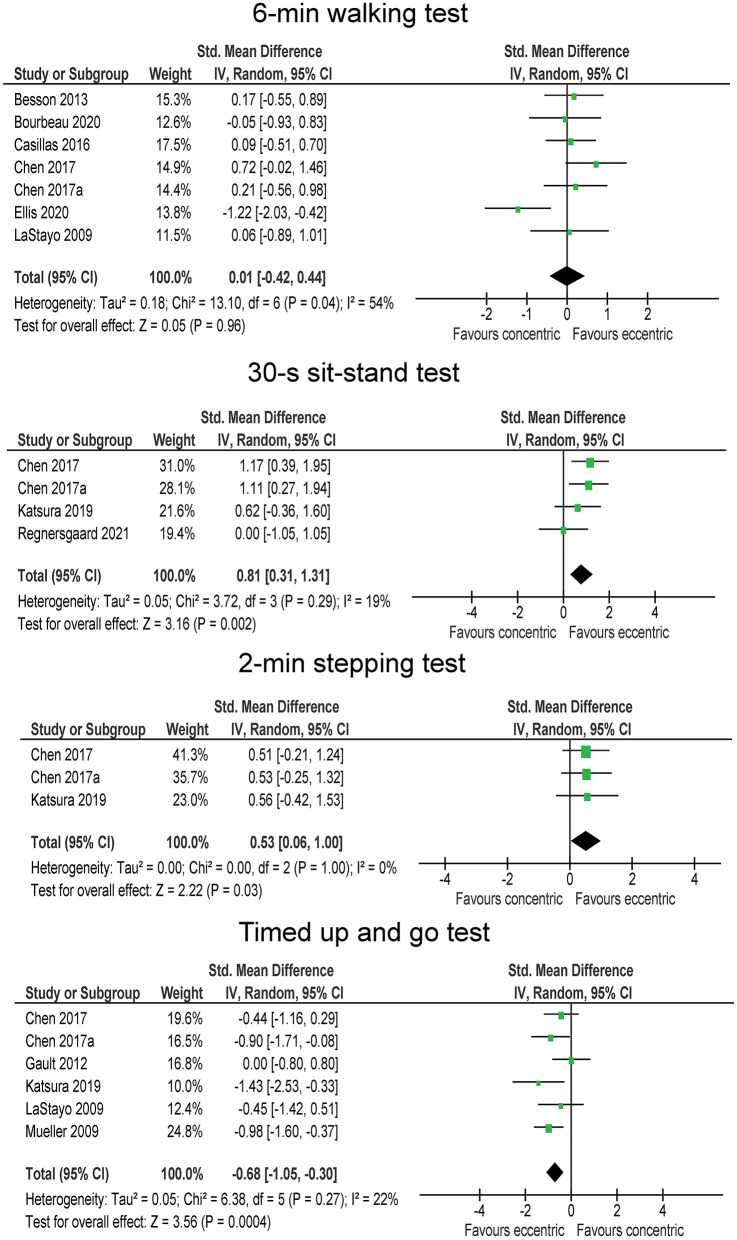
The effect of eccentric vs. concentric exercise on outcomes related to function and mobility.

The 6-min walking test was performed in 7 studies, involving a total of 97 participants in each group. The studies indicate no difference between the intervention groups on 6-min walking test (SMD = 0.01; *p* = 0.960). The heterogeneity among the studies was moderate (*I*^2^ = 54 %). On the contrary, statistically significant (*p* = 0.002) moderate effect (SMD = 0.81) in favor of eccentric exercise was shown for the 30-s sit-stand test (Figure 4), which was tested in four studies, involving 44 participants in eccentric exercise groups and 43 participants in traditional resistance or concentric exercise groups. The heterogeneity among the studies was low (*I*^2^ = 19 %). Two studies (44 and 43 participants in eccentric and traditional resistance or concentric exercise groups) included the stair descent test and tended to support a somewhat favorable effect of eccentric compared to traditional resistance or concentric exercise (SMD = −0.46), but not reaching statistical significance (*p* = 0.179). The two studies were homogenous (*I*^2^ = 0%). Moreover, two studies (involving a total number of 19 participants in each group) reported the results of stair ascent test (Figure 4). The difference between the exercise modes was trivial (SMD = 0.03; *p* = 0.930). Three studies reported on the 2-min stepping test results (37 participants in eccentric exercise groups, 36 in traditional resistance or concentric exercise groups) (Figure 4). The overall effect favored the eccentric exercise (SMD = 0.53) and was statistically significant (*p* = 0.03). The studies reported very consistent results, as shown by negligible heterogeneity (*I*^2^ = 0 %). TUGT was included in six studies (82 and 78 participants in eccentric and traditional resistance or concentric exercise groups, respectively). Similar to 30-s sit-stand, the results for TUGT were also statistically significantly (*p* = 0.004) better after eccentric exercise than traditional resistance or concentric exercise interventions (SMD = −0.68), and the heterogeneity among the studies was low (*I*^2^ = 22 %).

## Discussion

The purpose of this systematic review with meta-analysis was to examine the effects of eccentric exercise interventions in contrast to traditional resistance or concentric exercise interventions on muscle strength, body composition, muscle architecture, mobility, and function in older adults. We included 18 interventional clinical trials that had at least two intervention groups (eccentric and traditional resistance or concentric intervention group). The main findings of our systematic review are as follows: (a) for muscle strength, the pooled effect favored eccentric exercise, but the difference to traditional resistance or concentric was not statistically significant; (b) the results regarding body composition and muscle architecture were unclear and pooled from a smaller number of studies; (c) for mobility and function, the results were diverse, with the effects either in favor of eccentric exercise, or showing no difference between the intervention groups. In sum we examined a wide variety of tests and yielded different results. Because of this fact we cannot say with absolute certainty that eccentric exercises are superior to traditional resistance or concentric exercises and therefore cannot entirely reject nor accept our hypothesis. Although the results support the somewhat favorable effect of eccentric exercises, there are still a few questions that remain open.

The studies examined a wide variety of functional tests, including tests that emphasize aerobic capacity (6MWT, 2-min stepping test) and tests that assess functional performance and mobility (TUGT, 30-s sit-stand test, stair descending and stair ascending). Seven studies examined the effect of eccentric exercise on 6MWT. All the studies were in agreement that the effect of eccentric resistance training is similar to that of traditional resistance or concentric exercise. Some studies have speculated that the improvements in eccentric training are due to an increased contribution of anaerobic metabolism during eccentric training and a consequent improvement of its capacity. Adaptation of anaerobic capacity would particularly benefit shorter functional tests, such as 30-s sit-stand test (Besson et al., 2013; Laroche et al., 2013). On the other hand, it has been proposed that involvement of elastic components in eccentric exercise could improve movement efficiency, which is associated with reduced metabolic cost (LaStayo et al., 1999; Lindstedt et al., 2001) and could contribute to improvement in all functional tests. Namely, as eccentric exercise is associated with lower metabolic costs, it presumably enables higher force and power outputs to be reached during training, which would in turn elicit higher adaptations in muscle capacity. Even within endurance tests, such as 6MWT, greater anaerobic capacity could improve the performance, as resistance exercise interventions have been shown numerous times to improve economy of endurance activities (Hartman et al., 2007; Hunter et al., 2021). The 2-min stepping test was improved for ~10% in the eccentric exercise groups and ~5% in traditional resistance or concentric exercise group (Chen et al., 2017a,b; Katsura et al., 2019). Thus, both interventions improved the 2-min stepping test results, but the eccentric training group was even more effective (SMD = 0.53).

The TUGT and the 30-s sit-stand test are the among the most frequently used and scientifically supported tests for assessing overall function in older adults (Beauchet et al., 2011; Bennell et al., 2011; Barry et al., 2014). We found greater improvement in TUGT with eccentric exercise (*p* = 0.004; SMD = −0.68) compared to traditional resistance or concentric exercise. That was also the case with 30-s sit to stand test (*p* = 0.002; SMD = 0.81). However, it appears that the effects on maximal strength are similar in both groups although there was some tendency for larger effects in eccentric groups. One of the most common issues in older adults is sarcopenia, which is associated with a decreased quality of life, lower exercise tolerance and decreased levels physical activity levels in general (Maltais et al., 2014). Therefore, increasing or maintaining muscle mass and muscle strength need to be considered as a priority when planning training protocols for older adults. It is well-documented that resistance exercise is a very effective low-cost tool for preventing and treating sarcopenia and chronic diseases (Booth et al., 2012; Pedersen and Saltin, 2015; Ciolac and Rodrigues-da-Silva, 2016). As we can see from this systematic review, eccentric exercise can be as effective or even more effective than traditional resistance or concentric exercise. Of note, care should be taken when prescribing the quantity and intensity of exercise to avoid or minimize the risk of muscle damage and associated muscle soreness. Clos et al. (2021) came to a conclusion that when exercises are performed at the same power output, eccentric cycling elicits less intense perceptions of effort and muscle pain than concentric cycling. Maximal voluntary torque on the other hand showed a similar decline. When clinicians base the eccentric exercises on the rating of perceived exertion they should be very careful, given that most participants exhibit more neuromuscular performance decline after eccentric exercises (Clos et al., 2021).

The interventions considered in this review included either high-load eccentric resistance exercises, or low-load cyclic tasks performed in eccentric conditions or with accentuated eccentric portion of the movement (downhill walking, stair descending, and eccentric cycling). Downhill or downstairs walking is emerging as an effective type of eccentric exercise for the elderly. Assuming that training protocol is carried out where an elevator or any kind of assistance that helps the trainee to ascend is available, the intervention requires little additional equipment. Chen et al. (2017a) reported that descending stair walking improved muscle function, physical fitness, balance, cardiorespiratory fitness, lipid profiles, bone mineral density, and insulin sensitivity more than ascending stair walking in elderly obese women. In addition to providing great health and fitness benefits, downhill/downstairs walking is also metabolically less demanding than uphill/upstairs walking (Theodorou et al., 2013; Chen et al., 2017b; Regnersgaard et al., 2021). There is a wide variety of other means to perform eccentric exercise, such as working on resistance-training machines, performing everyday tasks in a way to emphasize eccentric contraction (e.g., sitting slowly down on a chair), or working on an eccentric cycle-ergometer. As said, one of the most important tasks of clinicians is that they dose the exercise correctly and that exercise is personalized by monitoring the rating of perceived exertion. Across the studies included in this review, the exercise was progressed gradually as fitness improved; either by pre-determined progression plan, or by tracking the rating of perceived exertion. Studies in this review mostly used the intensity corresponding to “somewhat hard” to “hard” exertion (between 9 and 13) on a Borg scale.

There are also a few limitations of this review to consider. The interventions were relatively short-term (6–12 weeks), which means that the differences between eccentric and traditional resistance or concentric exercise regarding long-term effects in older adults are not known. Moreover, several outcomes in the meta-analyses were pooled from a limited number of studies. In addition, subgroup analyses were not feasible due to the low number of studies. A few limitations also arise from the fact that there is sometimes hard to distinguish between ECC-only and ECC-emphasized exercise training protocols. It does raise an interesting question for further research on the topic, whether ECC-only training is more beneficial than merely emphasizing the ECC part of the exercise. Finally, the workload match between the exercise groups has to be questioned. Several studies used Borg scale, usually permitting some variation (e.g., 9–11 units). Some studies allowed participants to self-select the intensity (Gault and Willems, 2013) and very few used load prescription based on % repetition maximum (Reeves et al., 2009). It is not always clear how exercise programs should be matched to examine pure difference due to contraction type and maintain the ecological validity at the same time.

## Conclusion

In conclusion, our literature review indicates that there are a few tests in which eccentric training is superior or at least equal to traditional resistance or concentric training in maintaining health and overall function in older adults. The tests that showed the most effects for shorter (anaerobic) tests such as 30-s sit-stand test and TUGT. On the other hand, longer (aerobic) tests showed less improvements. When examining the papers, we also came to a conclusion that eccentric exercise is safe for frail and sick individuals. This type of exercise has to be well-planned and constantly monitored by clinicians. When progressing the exercise, one of the more useful tools to use is a combination of RPE, increasing quantity and a gradual increase in amount and intensity within a training protocol.

## Data Availability Statement

The original contributions presented in the study are included in the article/Supplementary Material, further inquiries can be directed to the corresponding authors.

## Author Contributions

KČ, JP, SL, ŽK, and NŠ conceptualized the idea. KČ and JP carried out the review and wrote the manuscript. NŠ and ŽK were overviewing the review procedure. NŠ, SL, and ŽK analyzed the collected data and finalized the manuscript. All authors contributed to the article and approved the submitted version.

## Funding

We want to acknowledge the support of the European Regional Development Fund and Physiko- and Rheumatherapie Institute through the Centre of Active Ageing project in the Interreg Slovakia–Austria cross-border cooperation program (partners: Faculty for Physical Education and Sports, Comenius University in Bratislava: Institute for Physical Medicine and Rehabilitation, Physiko- and Rheumatherapie GmbH). NŠ and ŽK acknowledge the University of Primorska's suppirt through internal research program KINSPO (2990-1-2/2021). The funders had no role in study conceptualization, data acquisition, data analysis or manuscript preparation.

## Conflict of Interest

NŠ was employed by company S2P, Science to Practice, Ltd. The company hand no role in conceptualization of the study, data acquisition, article writing nor any other phase of the study. The remaining authors declare that the research was conducted in the absence of any commercial or financial relationships that could be construed as a potential conflict of interest.

## Publisher's Note

All claims expressed in this article are solely those of the authors and do not necessarily represent those of their affiliated organizations, or those of the publisher, the editors and the reviewers. Any product that may be evaluated in this article, or claim that may be made by its manufacturer, is not guaranteed or endorsed by the publisher.
